# Bevacizumab-Induced Cutaneous Lupus Erythematosus in a Patient With Metastatic Colon Carcinoma: A Case Report

**DOI:** 10.7759/cureus.56559

**Published:** 2024-03-20

**Authors:** Sethi Ashish, Moses Raj, Eric Zhuang

**Affiliations:** 1 Infectious Disease, University of Missouri Kansas City School of Medicine, Kansas City, USA; 2 Hematology and Oncology, Allegheny Health Network, Pittsburgh, USA; 3 Hematology and Medical Oncology, Allegheny Health Network, Pittsburgh, USA

**Keywords:** drug-induced lupus, cetuximab, cutaneous lupus erythematosus, metastatic colon cancer, bevacizumab

## Abstract

Bevacizumab, an anti-vascular epidermal growth factor inhibitor, is approved for the treatment of various cancers. Hypertension, gastrointestinal perforation, bleeding manifestations, impaired wound healing, and cerebrovascular accidents are common side effects associated with the monoclonal antibody. Uncommon cutaneous reactions like exfoliative dermatitis associated with bevacizumab have been documented in the medical literature. We present an unusual case of bevacizumab-induced cutaneous lupus in a patient with metastatic colon cancer that started resolving after discontinuing chemotherapy. Timely intervention was key in preventing the progression of this chemotherapy-induced cutaneous lupus.

## Introduction

Bevacizumab is a recombinant, humanized monoclonal antibody that binds to vascular endothelial growth factor (VEGF) and is commonly used in combination with other chemotherapies for the treatment of various cancers, such as metastatic colorectal cancer, non-small cell lung cancer, ovarian cancer, and cervical cancer. Several trials of bevacizumab in the treatment of colorectal cancer have identified proteinuria, hypertension, delayed wound healing, arterial thromboembolism, and gastrointestinal perforation as common adverse effects [1]. We present an unusual case of bevacizumab-induced cutaneous lupus in a patient with metastatic colon cancer being treated in combination with folinic acid, fluorouracil, and irinotecan (FOLFIRI).

## Case presentation

**Figure 1:**
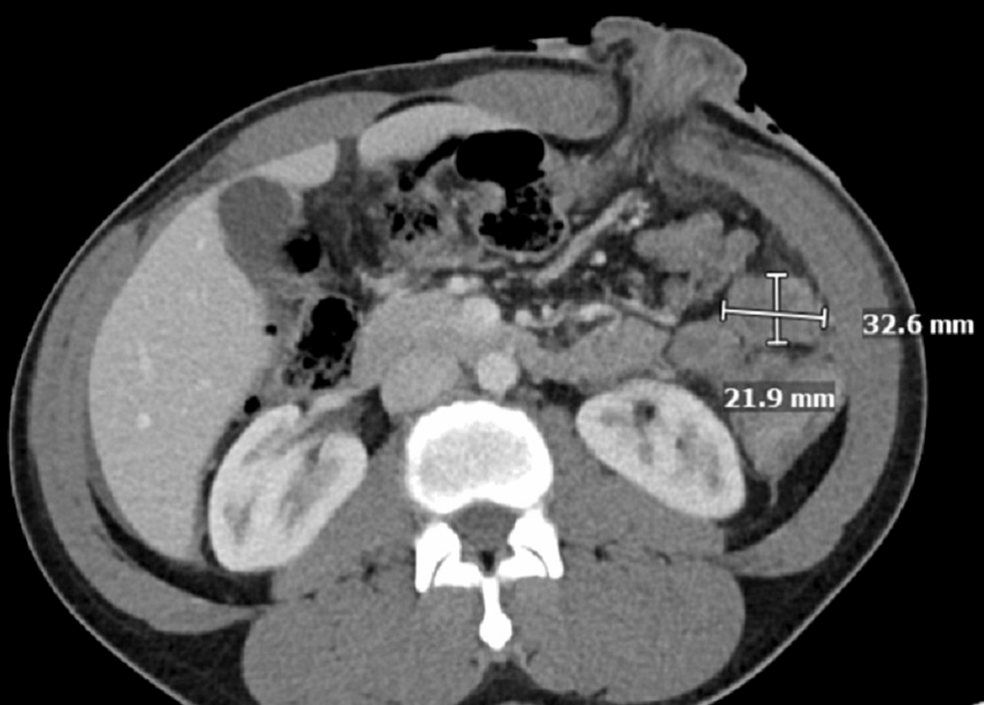
CT of the abdomen: before treatment Large splenic flexure mass with hepatic metastases. CT: computed tomography

**Figure 2:**
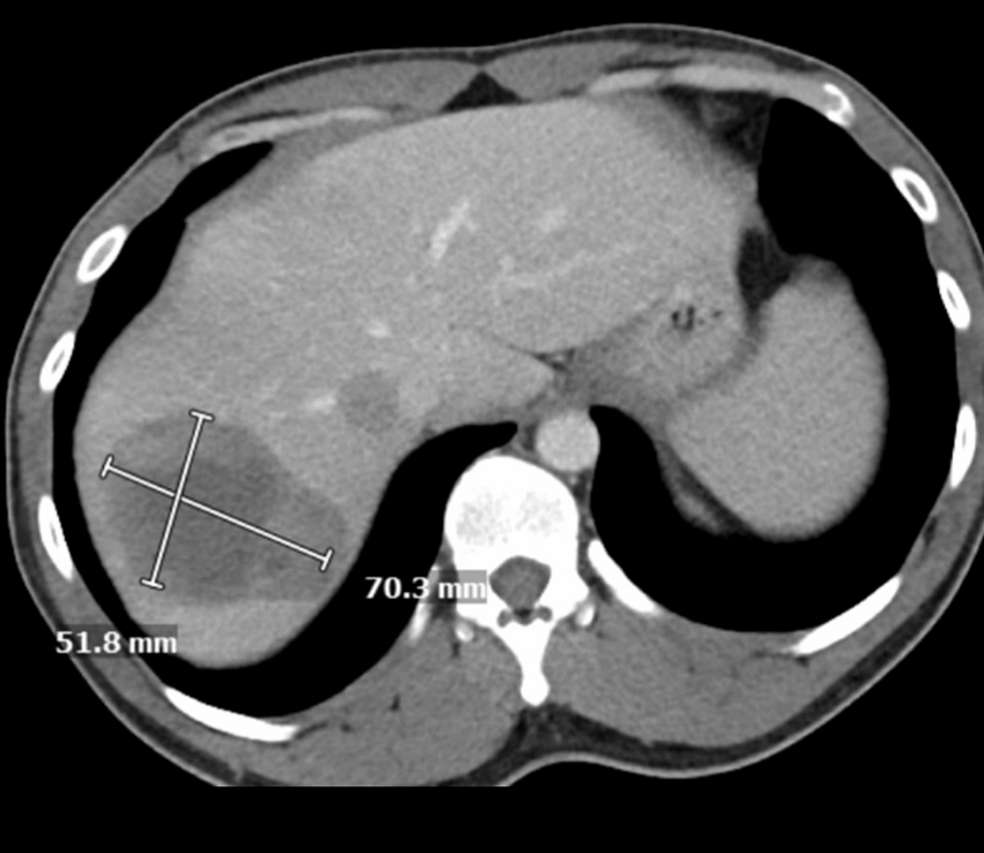
CT scan of the abdomen: before treatment Large multiple hepatic metastases were seen. CT: computed tomography

A 35-year-old male was admitted with complaints of acute abdomen pain, which was associated with nausea and vomiting. CT of the abdomen and pelvis revealed a large splenic flexure mass with the extraluminal extension of the mass and adjacent soft tissue (Figure 1). Also, numerous large hepatic masses were evident on the CT scan, illustrating metastases (Figure 2). The patient underwent a diverting loop transverse colostomy and liver biopsy, which confirmed stage IV metastatic colorectal adenocarcinoma.

**Figure 3:**
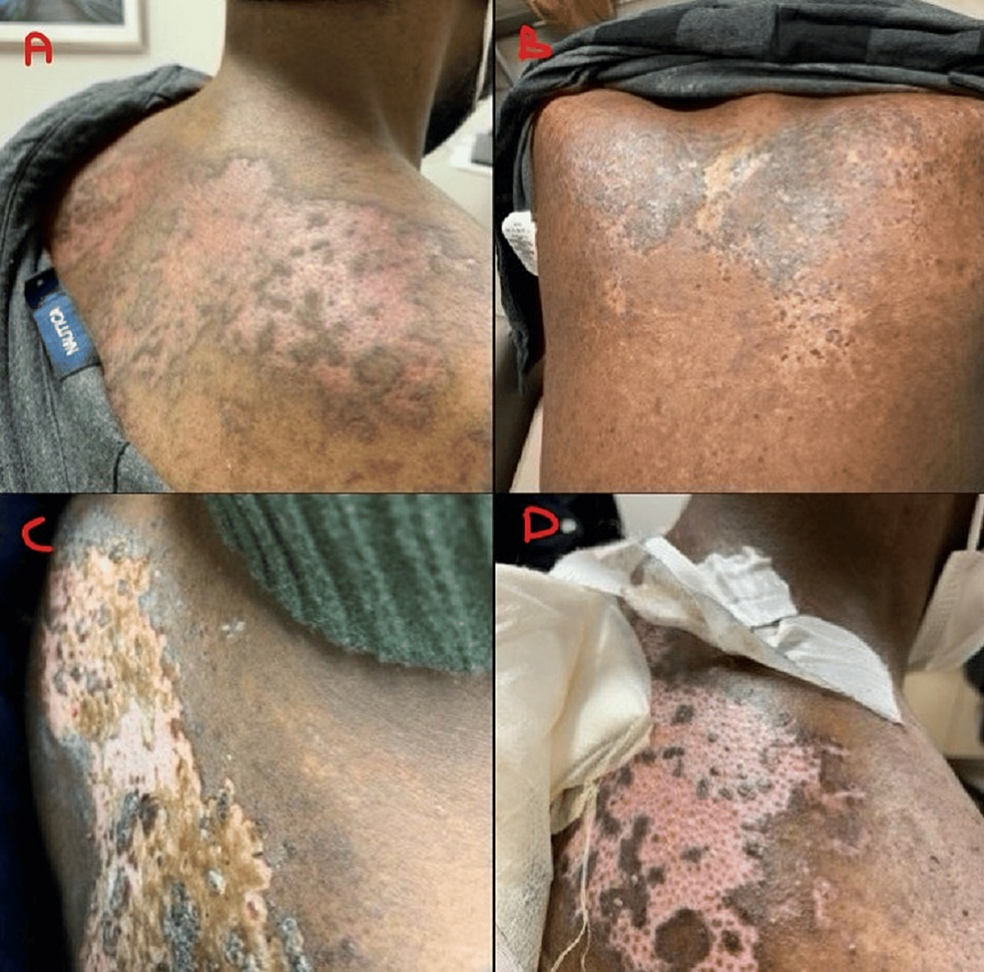
Post bevacizumab therapy: cutaneous lupus (A) discoid lupus erythematosus over the posterior trunk (torso); (b) discoid lupus erythematosus over the posterior trunk with scaling; (C) tender discoid lupus erythematosus over the posterior trunk with skin desquamation; (D) discoid lupus erythematosus over the posterior trunk with scaling skin desquamation and granulation tissue.

**Figure 4:**
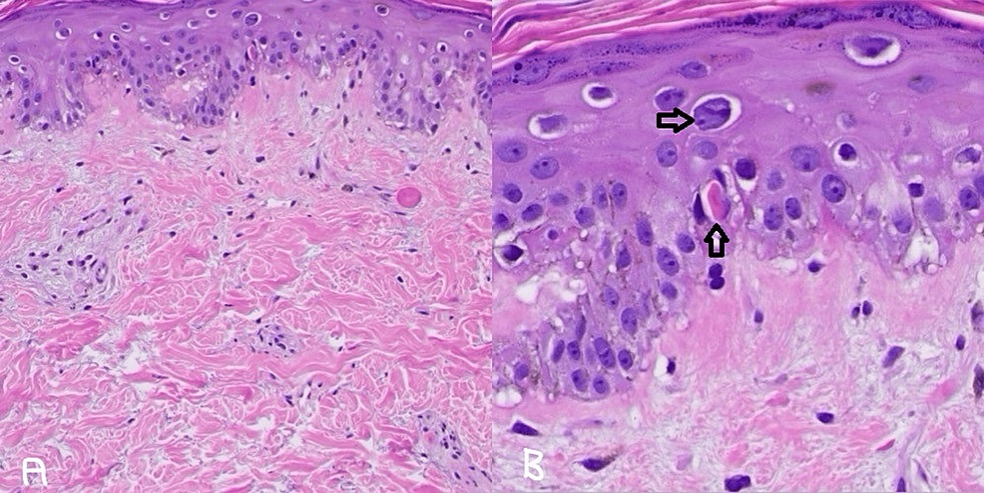
Histopathology/hematoxylin and eosin stain: cutaneous lupus erythematous (A) histologically subtle basal vacuolar interface dermatitis with increased interstitial dermal mucin; (B) side black arrow: basal vacuolization with lymphocyte satellitosis. Upward black arrow: rare dyskeratotic keratinocytes.

**Figure 5:**
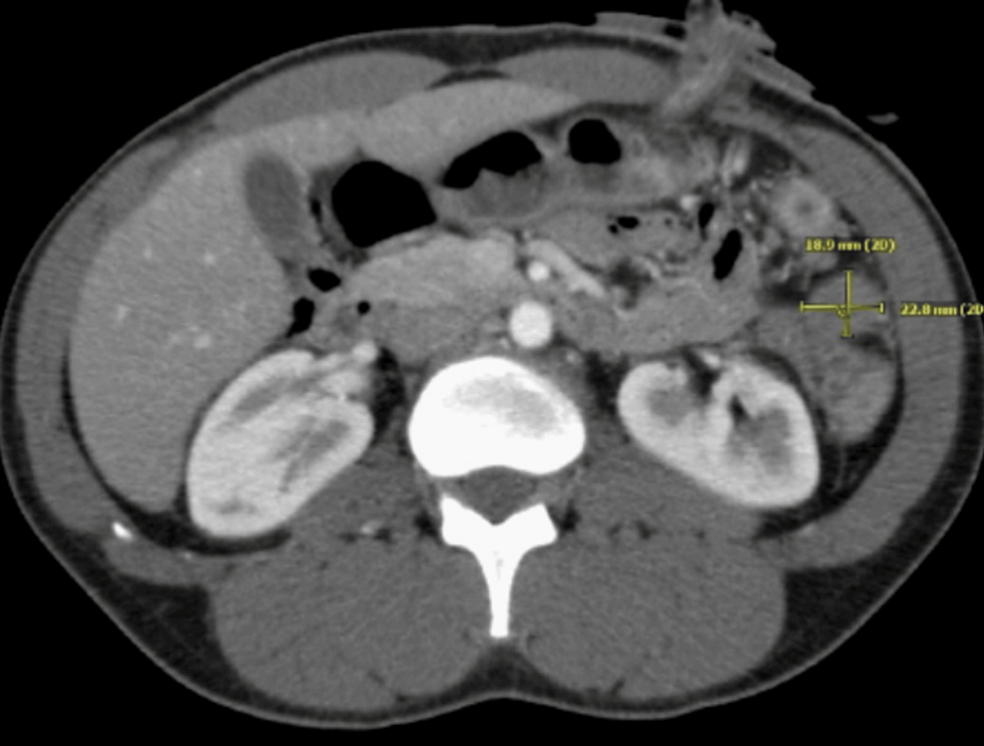
CT of the abdomen: after treatment Interval decrease in size of splenic mass with adjacent peritoneal disease CT: computed tomography

**Figure 6:**
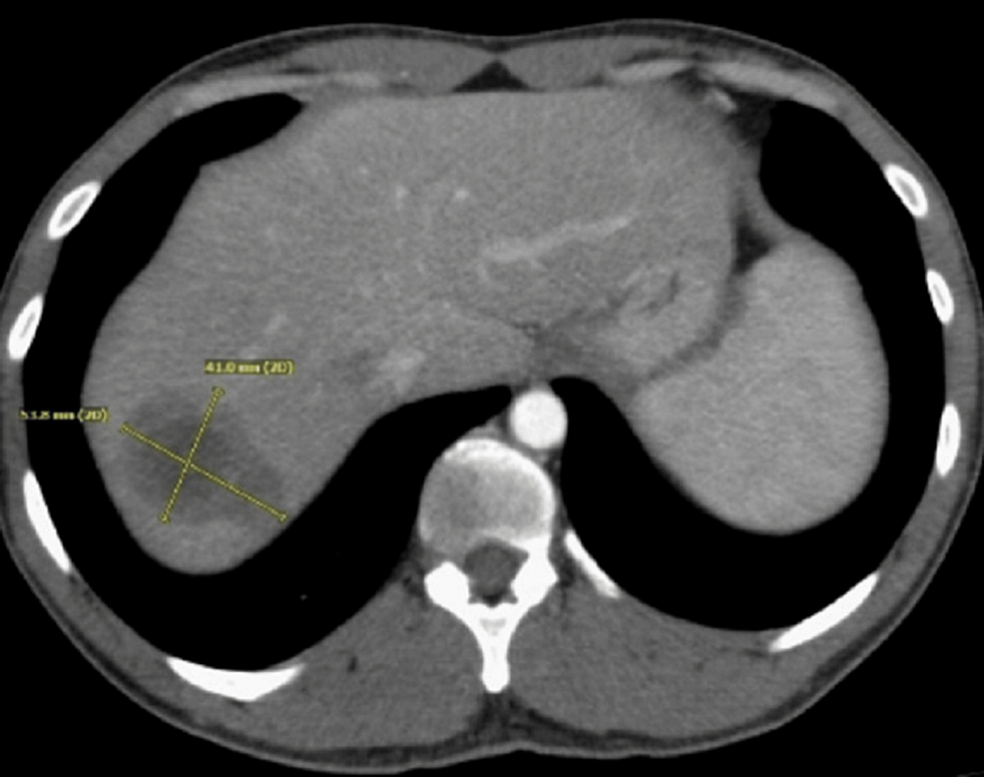
CT of the abdomen: after treatment Decrease in size of hepatic metastasis CT: computed tomography

The patient was initially treated with FOLFIRI for the first cycle of chemotherapy. Oxaliplatin was added to his second cycle of chemotherapy due to his excellent tolerance. By the time the patient was due for his fifth cycle of chemotherapy, next-generation sequencing revealed the patient's tumor was Kirsten rat sarcoma viral oncogene homolog wildtype; therefore, cetuximab was added with subsequent cycles of modified folinic acid, fluorouracil, irinotecan, and oxaliplatin (FOLFIRINOX). After receiving five cycles of a combination of FOLFIRINOX and cetuximab, the patient developed myositis due to paraneoplastic myopathy, which later resolved after discontinuation of cetuximab. Given the initial concern that cetuximab had contributed to his myositis while the paraneoplastic antibody panel was pending, the biological agent was changed to bevacizumab in combination with FOLFIRI for his resumptive cycle of treatment. On day 10 of this bevacizumab-containing cycle, the patient's wife noticed skin discoloration forming on the patient's upper back. However, he was otherwise asymptomatic at that time, so neither he nor his wife reported this to the medical team for evaluation. A few days later, on day 13, the patient presented in the clinic as the rash became scaly, itchy, and painful, with some areas peeling over his trunk (Figure 3a-3b). There were no signs of mucosal surface involvement, which lowered the suspicion of Stevens-Johnson syndrome or toxic epidermal necrolysis. Due to reports of exfoliative dermatitis from bevacizumab, this drug was held for his next cycle. Despite local therapy with topical corticosteroids, antihistamines, and silver sulfadiazine, his rash continued to worsen, became increasingly painful, and began desquamating a week later from the clinic evaluation, about 20 days after initially receiving bevacizumab (Figure 3c-3d). A skin punch biopsy demonstrated basal vacuolar interface changes accompanied by melanin incontinence and increased interstitial dermal mucin concerning drug-induced CLE (Figure 4). These findings, including the temporal association between his cutaneous eruption and bevacizumab administration, were highly suggestive of bevacizumab-induced CLE. Bevacizumab was therefore permanently stopped and was added to his drug allergy list. He was started on 1 mg/kg of oral prednisone. With a long steroid taper over several weeks, his symptoms improved, and his treatment was continued with the combination chemotherapy FOLFIRINOX, omitting bevacizumab. A restaging CT scan showed a decreasing size of his splenic flexure mass as well as a decrease in the hepatic metastases (Figures 5-6).

## Discussion

Cutaneous lupus erythematosus (CLE) is an autoimmune phenomenon that occurs spontaneously, in conjunction with systemic lupus erythematosus, or as a reaction to medication [2]. Drug-induced lupus erythematosus (DILE) is a lupus-like syndrome due to drug exposure that is usually resolved after discontinuation of the culprit drug [3]. DILE is most frequently reported in association with thiazide diuretics, calcium channel blockers, and antifungals and does not differ from idiopathic subacute cutaneous lupus erythematosus (SCLE) in its clinical and histopathological findings [2]. Drug-induced SCLE is the most common subtype of DILE and occurs several weeks to months after the initiation of the culprit drug. The onset of rash is typically in a photo-distribution and is associated with a positive serology to anti-Ro antibody [4]. The diagnosis of cutaneous DILE (CDILE) is usually based on clinical, pathological, and immunological features consistent with lupus erythematosus and on details of the disease history demonstrating a causal relationship with the suspected drug [3]. CLE in malignancies is mainly confined to immune-related adverse effects due to several immune checkpoint inhibitors (ICIs). As many drug-induced lupus resolves following discontinuation of the medication, ICI-induced subacute cutaneous lupus erythematous (SCLE) is a distinct entity, which can lead to delayed or prolonged skin reactions despite withholding the treatment [5].

However, a recent review documented an increase of 12.6% in the reporting of drug-induced lupus secondary to chemotherapies between 2009 and 2016, indicating that the incidence of lupus reactions due to chemotherapy is on the rise [6]. Among chemotherapies, VEGF inhibitors usually manifest as papulosquamous or polycyclic annular plaques, unlike exfoliated-type CLE, which our patient presented clinically. VEGF is an angiogenic mitogen that is expressed in activated macrophages and keratinocytes and is believed to play a major role in wound healing [7]. Vascular and endothelial cadherin repeats are relaxed by VEGF, which also controls vascular permeability and may be a causative factor leading to cutaneous erythematous rashes [8]. Several other chemotherapies, such as capecitabine, doxorubicin, and gemcitabine, have been associated with CDILE [9]. Docetaxel, an anti-microtubule class of chemotherapeutic agent, has also been reported to cause cutaneous lupus in the form of an erythematous desquamating rash during early-stage breast cancer [10]. Similarly, Tokunaga et al. reported five patients with stage IIIB and IVB ovarian cancer treated with bevacizumab and a docetaxel-carboplatin combination having skin desquamation or skin hardening followed by peeling [11]. Also, Shakhbazova and Marsch reported different subtypes of lupus erythematosus, and the majority manifested as cutaneous-like reactions induced by systemic administration of drugs like 5-fluorouracil, capecitabine, and uracil-tegafur [4]. No reports of CDILE in combination with FOLFIRINOX and bevacizumab have been reported in the medical literature. However, Bang et al. reported a case-control study of hand-foot syndrome in 58.6% of cases as a dermatological reaction following combination treatment with bevacizumab and capecitabine [12]. Further research is warranted to understand the pathogenesis and management of bevacizumab and other anti-angiogenesis-induced discoid lupus erythematosus.

## Conclusions

It is critical to biopsy skin lesions, look for evidence of other cutaneous side effects, and not just be confined to exfoliative dermatitis with bevacizumab. Also, withholding the drug is imperative. Oral corticosteroids may help with reducing symptoms and reducing the duration of cutaneous lupus associated with bevacizumab. Further research is required to manage this unusual side effect with bevacizumab.
